# Editorial: Parental influence on child social and emotional functioning

**DOI:** 10.3389/fpsyg.2024.1392772

**Published:** 2024-03-12

**Authors:** Xiaoqin Zhu, Diya Dou, Thanos Karatzias

**Affiliations:** ^1^Department of Applied Social Sciences, The Hong Kong Polytechnic University, Hunghom, Hong Kong SAR, China; ^2^School of Health and Social Care, Edinburgh Napier University, Edinburgh, United Kingdom

**Keywords:** parenting, family, social adjustment, child development, mental health, youth wellbeing

An individual with healthy social and emotional functioning can experience, express, and manage emotions well, form and sustain positive social relationships, and adapt to social contexts effectively, which is essential for a person's overall healthy functioning and mental health, especially among children and adolescents (Mahoney et al., 2021). Research suggests that children who have positive social-emotional health tend to be happier, demonstrate better academic performance, and display fewer problematic behaviors than less socially and emotionally competent peers (Malti and Song, 2018; Collie, 2022; Guo et al., 2023). Such powerful effects are long-lasting and can extend from early years to adulthood (Luecken et al., 2013).

Worldwide, there is a consensus that social and emotional development is a result of individual-context interactions (Lerner and Castellino, 2002). Children learn and practice their social-emotional skills in social interactions with parents, peers, and teachers. Particularly, socialization perspectives regard parenting as the primary factor that shapes child and adolescent development to a large extent (McDowell et al., 2002). Despite that a large number of empirical studies have linked different parenting practices to child and adolescent social-emotional development, the underlying psychological and behavioral mechanisms are still largely unknown, especially with regard to different age groups (e.g., young children and adolescents) and different contexts (e.g., rural-urban or cultural disparities). Another neglected area of research relates to how parental mental health may affect children's outcomes and in what ways. Thus, this Research Topic aims to enrich the existing literature in the field by welcoming submissions from different disciplines (e.g., psychology and social work) that may employ different research methods, such as quantitative, qualitative, systematic review, and conceptual studies.

Early interactions with parents are critical for children to understand oneself, others, and the world. Parents may have different socialization practices, thus introducing different developmental trajectories to children (Kochanska et al., 2019). A nurturing, encouraging, and warm family environment often reflects healthy parent-child relationships that promote positive development and adjustment among young children; on the contrary, a family context characterized by stress, neglect, rejection, or dysfunctions would put children at risk for maladjustment later in life (Krauss et al., 2020; Ward and Lee, 2020; Zhu and Shek, 2021; Chen et al., 2022). In this Research Topic, six articles enriched the literature by discussing parenting practices and parental influences among preschool children.

King et al. reported three latent profiles of parental emotion socialization processes in terms of emotion coaching, emotion dismissing, and emotion disengagement among Western parents of children aged 4–10 years. The finding empirically supports emotion socialization theories that advocate differentiated classification of emotion coaching (i.e., parents validate and teach children about emotions) and emotion-dismissing (i.e., parents minimize and dismiss their children's emotions) in parenting. Future research should focus on how these distinct parental emotional socialization strategies would affect children's development.

Li and collaborators illustrated how parenting styles were associated with child development among Chinese preschool children in two articles. The article by Li et al. (a) entitled “*The association between authoritarian parenting style and peer interactions among Chinese children aged 3–6: an analysis of heterogeneity effects”* reported the negative effects of authoritarian parenting on children's peer interactions. Such effects were stronger among boys, younger children, or children with siblings. The second article by Li et al. (b) entitled “*Parenting style and children emotion management skills among Chinese children aged 3–6: the chain mediation effect of self-control and peer interactions”* further described the negative and positive predictive effects of authoritarian and authoritative parenting styles, respectively, on children's emotion management skills through the chain medication effects of children's self-control and peer interactions. Also focusing on Chinese preschool children, Zhao et al. found that parents with high parenting stress were less likely to exercise authoritative parenting, which hindered preschoolers' learning approaches. In addition, compared to migrant children, native children benefited more from an authoritative parenting style.

Chen further investigated how parental mental health was associated with child development among preschool Chinese children. The results showed that parental depression was likely to increase parental stress and child maltreatment, which in turn jointly raised the risk of child internalizing and externalizing problems. The study by Jiang et al. showed the long-term effect of parental absence in early years. They found that individuals living without parents during childhood and adolescence reported worse physical and mental health status in adulthood, regardless of age and gender.

Findings from these studies support the essential and long-lasting influence of interactions between children and their parents in their early years. In short, parents' mental health and wellbeing may to some extent determine their parenting practices, such as emotional socialization approaches, and authoritarian or authoritative parenting styles, which may subsequently influence their young children's social, emotional, and behavioral adjustment. Such pathways are likely to hold in different contexts despite stronger effects among certain groups (e.g., boys).

When children enter adolescence, their development is characterized by a growing need for independence from parents and expanding social interactions beyond family. Nevertheless, parenting and parent-adolescent relationships still serve as a significant shaping force in adolescent development (Zhu and Shek, 2021; Zhou et al., 2024). Nine studies included in this Research Topic demonstrate significant parental effects among adolescents in different cultures.

First, three studies revealed the effect of overall family environmental factors on Chinese adolescents and explored mediation and moderation pathways. Zhou et al. reported on the relationship between family intimacy and peer relationships among Chinese adolescents with psychological capital and self-identity serving as a mediator and a moderator, respectively. Specifically, a higher level of family intimacy was significantly associated with adolescents' stronger psychological capital, which in turn led to better peer relationships. Furthermore, such associations were stronger among adolescents with stronger self-identity. Cui et al. found that family function was a positive predictor of adolescent altruistic behavior through the chain mediation effect of self-affirmation and psychological resilience. Lai and Chen found that family cohesion and adaptability were negative predictors of depression, which positively predicted non-suicidal self-injury (NSSI) among adolescents. Further, the association between depression and NSSI was mitigated by school connectedness, highlighting the protective effect of ties between adolescents and the school.

Second, six articles, including one meta-analysis and one conceptual article, focused on specific parental factors. Habibi Asgarabad et al. demonstrated the positive link between parental psychological control as an intrusive parenting behavior and adolescents' externalizing (e.g., aggression and rule-breaking) and internalizing (e.g., depression and withdrawal) problems in Iran by firstly establishing psychometric properties of a Persian version of the Psychological Control Scale-Youth Self-Report (PCS-YSR). Pan et al.'s meta-analysis reviewed 35 studies and identified a significant positive relationship between parents' use of corporal punishment and a spectrum of violent behaviors among adolescents such as aggression, anti-social behavior, and criminal behavior, with a small to medium pooled effect size. The effect was independent of developmental stage (childhood vs. adolescence vs. adulthood), gender, culture, and type of violence. However, the association was stronger when corporal punishment was more severe.

Ratliff et al. reported significant positive links between supportive parent-adolescent relationships and positive adjustment among American adolescents in terms of more prosocial behavior, less aggressive behavior, and fewer depressive symptoms, which were significantly mediated by adolescent emotional regulation. Such pathways were more salient among adolescent boys in comparison to adolescent girls. Boullion et al. also reported the mediation effect of emotional regulation in linking parental factors and developmental outcomes among adolescents in the United States. They found that perceived parental warmth and affection at age 12 predicted adolescents' reduced use of expressive suppression emotion regulation strategy at age 14, which further predicted decreased internalizing problems from age 14 (before the pandemic) to age 15 (the initial phase of the pandemic in Spring 2020 in the United States).

Long et al. provided evidence for the importance of social relationships and social-emotional development in adolescents' overall health and wellness through an evaluation study using a randomized controlled trial design. They found that a 15-week school-based universal mental health education intervention aiming to promote students' healthy living such as self-awareness, emotional health, meaning, and social relationships in contexts of family, peers, and community effectively enhanced self-confidence and life satisfaction among early adolescents (grades 4–6) in the experiment group.

The conceptual article by Rivas and Albertos proposed a novel conceptual framework linking adolescents' experience of optimal frustration that can be facilitated by positive family leisure time and the development of adolescent autonomy. Grounded in Self-Determination Theory and other related theoretical notions such as the theory of flow, the authors argued that although feelings of frustration frequently experienced by adolescents may lead to unfavorable outcomes, it can also provide adolescents with opportunities to learn and practice skills for overcoming adversity, thus fostering resilience and socioemotional wellbeing. The authors also discussed why and in what ways such optimal frustration can be experienced during positive family leisure time. Specifically, through spending quality leisure time together with adolescents, which is characterized by emotional support, communicative interactions, active parental involvement, and positive parental supervision, parents can create a conducive and nurturing family environment where adolescents have opportunities to experience positive frustration while also receiving support, encouragement, guidance, and resources to tackle challenges effectively. To sum up, the authors' conceptual discussion advances our understanding of the role played by parents in adolescent development from a unique perspective and it encourages further research in this area.

All this work with young children and adolescents further reinforces the continuous parental influence on child and adolescent social, emotional, and behavioral development, which may be operated by multiple mediation pathways and interplay with different personal (e.g., adolescents' self-identity) and contextual factors (e.g., school). In addition, these pathways can be utilized in prevention and intervention programs to facilitate children's and adolescents' overall healthy development.

The last article by Matthewson et al. presents findings of a qualitative study on the experiences of voluntary reunification based on interviews with both adult alienated children and targeted parents. Six themes were identified based on sharing from adult alienated children (e.g., factors influencing reunification and the effect of communication) and three themes from narratives provided by the targeted parents (e.g., understanding of reunification and life after reunification). The authors concluded that voluntary reunification is a long-term process that may last for decades and include periods of connection and rejection.

We hope that the studies included in this Research Topic will enable researchers in different fields (e.g., education, psychology, or social work) to exchange both theoretical discussions and empirical practices regarding the relationship between parental factors (e.g., overall familial environment and specific parenting practices) and the child's social and emotional development at different stages (e.g., preschool and adolescence). The Research Topic also delineates different mediation and moderation mechanisms that underlie parental influence on child and adolescent social-emotional adjustment. Findings from all this work provide insights into effective practices for both parenting and youth programs to promote social and emotional functioning among children and adolescents, such as fostering effective emotional socialization and enhancing emotional regulation strategies among children.

It should be noted that interactions between children and parents are bi-directional, meaning they affect each other (Lerner and Castellino, 2002; Zhu and Shek, 2020). Parenting can be parents' active socialization actions that influence their children's development (i.e., parent effect); it can also be parents' reactions to their children's social and emotional functioning (i.e., child effect). Nevertheless, the Research Topic has not included any studies pertinent to the child effect. More effort needs to be devoted to this research area. In addition, only one article (i.e., Lai and Chen) in the Research Topic explores the interplay between family and school factors. Future research should explore factors that may affect child and adolescent social-emotional development in different socialization contexts (e.g., family, school, and culture) simultaneously.

## Author contributions

XZ: Writing – review & editing, Writing – original draft. DD: Writing – review & editing. TK: Writing – review & editing.
